# A New Method for Preparation of Decellularized Human Scaffolds for Facial Reconstruction

**DOI:** 10.3390/cimb47040275

**Published:** 2025-04-14

**Authors:** Elise Lupon, Aylin Acun, Alec R. Andrews, Ruben Oganesyan, Hyshem H. Lancia, Laurent Lantieri, Mark A. Randolph, Curtis L. Cetrulo, Alexandre G. Lellouch, Basak E. Uygun

**Affiliations:** 1 Department of Plastic and Reconstructive Surgery, Institut Universitaire Locomoteur et du Sport, Pasteur 2 Hospital, University Côte d’Azur, 06107 Nice, France; elise.lupon@gmail.com; 2 Laboratory of Molecular PhysioMedicine (LP2M), UMR 7370, CNRS, University Côte d’Azur, 06107 Nice, France; 3 Vascularized Composite Allotransplantation Laboratory, Center for Transplantation Sciences, Massachusetts General Hospital, Harvard Medical School, Boston, MA 02115, USA; 4 Shriners Children’s Boston, Boston, MA 02114, USA; aacun@widener.edu (A.A.);; 5 Center for Engineering in Medicine and Surgery, Massachusetts General Hospital, Harvard Medical School, Boston, MA 02114, USA; 6 Department of Biomedical Engineering, Widener University, Chester, PA 19013, USA; 7 Department of Pathology, Beth Israel Deaconess Medical Center, Harvard Medical School, Boston, MA 02215, USA; 8 TIMC Lab, University of Grenoble Alpes, French National Centre for Scientific Research (CNRS), UMR 5525, 38000 Grenoble, France; 9 Service de Chirurgie Plastique et Reconstructrice, Hôpital Européen Georges-Pompidou, 75015 Paris, France; laurentlantieri@gmail.com; 10 Faculté de Médecine, Université Paris Cité, 75006 Paris, France; 11 Department of Plastic and Reconstructive Surgery, Massachusetts General Hospital, Boston, MA 02114, USA; 12 Division of Plastic and Reconstructive Surgery, Cedars-Sinai Medical Center, Los Angeles, CA 90048, USA; 13 Innovative Therapies in Haemostasis, INSERM UMR-S 1140, University of Paris, 75006 Paris, France

**Keywords:** decellularization, recellularization, vascularized composite allotransplantation, facial reconstruction, regenerative medicine, scaffold, tissue engineering

## Abstract

Vascularized composite allotransplantation (VCA) has emerged as a robust alternative for addressing anatomically complex defects but requires a toxic lifelong immunosuppressive regimen. Tissue engineering offers the promise of creating recipient-specific alternative grafts using a decellularization and recellularization approach. In this article, we establish a reliable protocol for human face decellularization by immersion as a new tool in the development of engineered graft alternatives for reconstructive surgery. Three cadaveric face grafts were immersed in 1% sodium dodecyl sulfate for 216 h followed by 1% Triton X-100 for 48 h, without perfusion through the pedicle. We determined that decellularization was successfully accomplished for three facial specimens as confirmed by histological evaluation and quantification of DNA content. The extracellular components including collagen, glycosaminoglycans, elastin, and matrix-bound growth factors were preserved. Vascular architecture did not show significant differences between native and decellularized grafts as imaged by X-ray angiography. The mechanical strength of the grafts was not altered after decellularization. We also showed that the decellularized grafts were biocompatible in vitro and in vivo allowing cell engraftment. As a result, we have successfully developed a protocol to yield a clinical size decellularized graft suitable for generating a recellularized, potentially non-immunogenic graft for facial reconstruction.

## 1. Introduction

Since 2005, facial vascularized composite allotransplantation (FVCA) has proven to be a groundbreaking reconstructive procedure for severe disfigurements [1,2]. However, the inclusion criteria for FVCA remain limited due to the requirement for lifelong immunosuppression and the potential risk of chronic rejection [3,4,5]. Recently, issues with chronic rejection have necessitated surgically complex re-transplantations [6], raising concerns about the evolution of these grafts and long-term therapeutic alternatives.

Tissue engineering provides the means to develop alternative tissues for VCA that have potential to be patient-specific and hence eliminate the need for immunosuppression. As a result, the risks associated with this life-changing procedure for patients could be significantly reduced [7,8]. Decellularization and recellularization represent a tissue engineering solution that has shown great potential in solid organ transplantation. However, less is known about their application in the context of composite tissue allografts [9]. Decellularization, which involves the removal of native cells from the tissue without disturbing the composition and the architecture of the extracellular matrix, is achieved by treating the tissue or organ with detergents, enzymes, or chemicals either by simple immersion or perfusion through the vasculature [10]. Once the native cells are removed, the patient-specific cells can be introduced into the decellularized tissue to make the construct viable and transplantable and potentially non-immunogenic [7,10].

Biological scaffolds, composed of decellularized extracellular matrices, have long been used in plastic surgery to repair various tissues, including the skin [11,12,13]. Yet, no decellularized and/or recellularized VCA has truly functioned as full-thickness skin [14] because of the need to functionally replace multiple tissue types in a single unit and the need for transplantation with microsurgical anastomosis. Preliminary work by Duisit et al. [15] has shown the feasibility of producing a matrix from human cadaveric face grafts using a pedicle perfusion system. However, other teams have suspected this way of decellularization to be a source of vascular lesions, which may cause transplantation and recellularization failures [16]. We already had conclusive experience of decellularization using the imbibition technique on small tissue surfaces, such as nipples [17], but its efficacy on larger tissue surfaces does not appear to be described in the literature.

In this study, we introduce a novel protocol for the decellularization of complex engineered grafts designed for facial reconstruction. This protocol involves the preparation of a decellularized human face through imbibition, offering the potential for subsequent repopulation with patient-specific cells. Furthermore, fundamental parameters, which have received less investigation in previous studies [15], such as the preservation of mechanical properties, specific growth factors essential to the functionality of the scaffolds, and the immunogenicity of the scaffolds, were evaluated.

## 2. Materials and Methods

### 2.1. Tissue Procurement

The study was conducted on four total face grafts procured from fresh human cadavers donated to the International Institute for the Advancement of Medicine (IIAM) (Edison, NJ, USA). The median duration from the time of death to tissue procurement was 32 h (range 24–48 h). The median age of the donors was 62 years (range 55–85). The cadaver heads were transported to the laboratory in a fresh state, without the use of fixation products and were stored in an ice bag at room temperature until fresh grafts were procured. All experiments were approved by Massachusetts General Hospital (MGH) Institutional Review Board (IRB).

Human facial grafts were procured by board-certified plastic surgeons at MGH. The soft tissues of the face, including muscles and their innervation were taken by dissecting the vessels at their origin at the external carotid artery and the internal and external jugular vein. The graft was raised from lateral to medial in a cranio-caudal direction. The cannulation of the right and left facial arteries and the right and left temporal arteries was performed while preserving veins. The grafts were flushed with heparinized saline (15 UI/mL, 500 mL) after harvesting. The checklist with the different steps of the surgical technique is available in Table A1.

### 2.2. Decellularization of Facial Grafts

**Figure 1:**
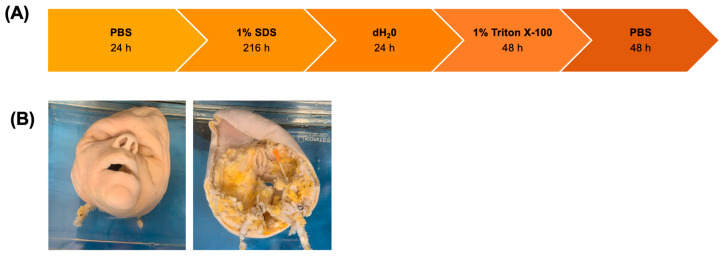
Decellularization of facial grafts. (**A**) Schematic of decellularization protocol. Solutions and immersion duration are indicated. (**B**) Facial graft in decellularization solution; left: epidermal; right: dermal.

Human facial grafts were immersed in a glass jar containing the decellularization solutions at room temperature. The decellularization solutions were changed following a protocol previously optimized for porcine fasciocutaneous flaps [18]. Briefly, the tissue was washed with phosphate-buffered saline (PBS) for 24 h, followed by 1% sodium dodecyl sulfate (SDS) for 216 h. After washing with deionized water (dH_2_O) for 24 h, the grafts were immersed in 1% Triton X-100 for 48 h and finally in PBS for 48 h (Figure 1). On day 2, de-epithelialization was performed mechanically with forceps. The grafts were monitored for edema, blistering of facial skin and mucosae, fat, and muscle bleaching during all steps. At the end of the protocol, scaffolds were either processed immediately for analysis or stored at 4 °C in PBS until further use.

### 2.3. Analysis of DNA Removal

The DNA content was analyzed for tissues sampled from skin, and cartilage (concha) and bone (clean nose bone) of native and decellularized grafts. Twenty-five milligram biopsy samples were collected from the center and periphery of the grafts and DNA was extracted using the DNeasy Blood & Tissue kit (Thermofisher Scientific, Waltham, MA, USA). The quantity of purified DNA from each sample was determined using the absorbance values at 260 nm measured by Nanodrop (Thermofisher Scientific, Waltham, MA, USA). The final value was expressed as weight of DNA per weight of wet tissue. One of the face grafts underwent daily sampling at different graft locations (skin and subcutaneous tissue) throughout the decellularization in order to monitor the DNA removal profile.

### 2.4. Quantification of Extracellular Matrix Components

Using a 3 mm surgical biopsy punch, native, fat, and skin samples were collected from the center and periphery of the decellularized and native grafts for each analysis. Total collagen content was determined using the total collagen kit (QuickZyme Biosciences, Leiden, The Netherlands). Glycosaminoglycan (GAG) content was analyzed using a colorimetric assay as described previously [18]. The grafts were also analyzed for any matrix-bound growth factors using RayBio^®^ Human Growth Factor Antibody Array G-Series 1 kit (RayBiotech Life, Peachtree Corners, GA, USA) following the manufacturer’s instructions.

### 2.5. Histological Assessment

Native and decellularized tissue samples were fixed in 10% neutral buffered formalin for 24 h, dehydrated, embedded in paraffin, and sectioned (5 μm) following standard protocols. The sections were stained with hematoxylin and eosin (H&E) for general tissue morphology, Masson’s Trichrome to highlight collagen, and Van Gieson’s stain to visualize elastic fibers. The whole tissue sections were scanned using Hamamatsu Nanozoomer Digital slide scanner (Hamamatsu Photonics K.K., Hamamatsu City, Japan).

### 2.6. Assessment of Vascular Architecture

A contrast agent (Visipaque, GE Healthcare, Chicago, IL, USA) mixed with normal saline (1:2) was injected into the arterial pedicle using constant syringe pressure for the native graft before decellularization and for the graft after decellularization (but before sample collection to preserve vascular integrity).

Image acquisition was performed with a Powermobil C-Arm (Siemens, Munich, Germany). Images were exported in DICOM format and visualized with Osirix MD software (Pixmeo, Bernex, Switzerland). This examination was performed on each of the facial grafts native and decellularized.

### 2.7. Scanning Electron Microscopy Imaging

Electron microscopy of native and decellularized facial grafts was performed at the Schepens Eye Institute core facility supported by the NIH National Eye Institute Core Grant #P30EY003790. The samples were prepared as follows: First, they were dehydrated in graded ethanol solutions and dried using a Samdri 795 critical point dryer (Tousimis, Rockville, MD, USA). Next, they were mounted on aluminum pedestals and chromed using a Gatan high-resolution ion beam coater (Gatan Inc., Pleasanton, CA, USA). Finally, the coated samples were imaged using a JEOL JSM-7401F field emission scanning electron microscope (JEOL Inc., Peabody, MA, USA), allowing for a qualitative assessment of the tissue architecture.

### 2.8. Mechanical Testing of Decellularized Grafts

**Figure A1:**
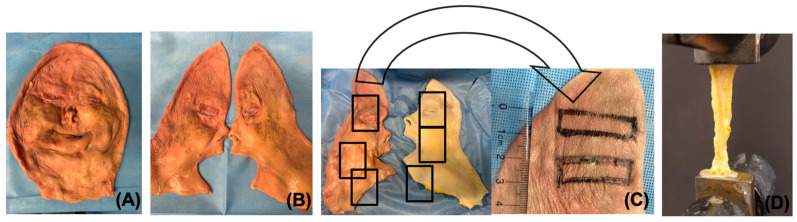
Tensile test procedure. (**A**) Native face. (**B**) Face split in two. One of the hemiface is decellularized. (**C**) Rectangular section of sample on each hemiface. (**D**) Tensile mechanism.

Tensile testing was performed on native and decellularized skin samples from facial grafts. Briefly, one face graft was cut in half along the forehead and chin axis (Figure A1A,B). One half was decellularized according to the protocol described above and the other half was kept under sterile conditions until analysis to serve as the native sample (Figure A1C). For testing, 4 × 1 cm^2^ rectangular sections were taken from three separate locations (e.g., forehead, cheek, and chin) of the decellularized and native half face grafts (Figure A1C). Each piece was subjected to a uniaxial tensile strength test using an Instron 5984 Universal Testing Machine (Instron, Norwood, MA, USA) until failure (Figure A1D). The strain was calculated as the ratio of total displacement over an initial grip length of 20 mm. The stress was calculated as force applied divided by sample area. The stress–strain curves were plotted, and they were used to determine the maximum tensile strength as the strength before sample failure and the Young’s modulus as the slope of the linear portion of the curve in Microsoft Excel. The resulting values for the native and decellularized samples from each location of the facial grafts were compared using multiple paired *t*-tests with Holm–Šídák method.

### 2.9. In Vitro Biocompatibility of Decellularized Grafts

Decellularized face samples were tested in in vitro cell culture for inducing cell proliferation and engraftment. First, the samples were cut into sections of 0.5 cm^2^ and incubated in sterile PBS supplemented with 4% ethanol and 0.1% peracetic acid for 48 h under agitation for sterilization. Next, the samples were placed in the wells of an ultra-low attachment 96-well plate either on the dermal side or on the epithelial side for cell seeding. Before the addition of the cells, the scaffolds were preconditioned with fibroblast base culture media supplemented with serum (ATCC, Manassas, VA, USA) at 37 °C, 5% CO_2_ for approximately 30 min. The cells that were used in the experiments were primary human dermal fibroblasts (ATCC, Manassas, VA, USA) cultured in basic fibroblast medium (ATCC, Manassas, VA, USA) supplemented with a low-serum fibroblast growth kit (ATCC, Manassas, VA, USA) until confluent. The cells were trypsinized and resuspended in culture medium for plating on the scaffolds at a density of 1 × 10^5^ cells per scaffold. The cell growth was monitored for 72 h by Presto Blue assay (Thermofisher Scientific, Waltham, MA, USA) performed daily according to the manufacturer’s recommendations. At the end of the 72 h culture, the scaffolds were fixed in 10% neutral buffered formalin and processed for histology, as described above for H&E staining. To determine the depth of cell penetration, 5 histological sections were selected and the distance of the farthest cells from the surface was determined using Image J (version 1.53c). A total of 75 measurements were performed per group. The average length of the cells from the surface of the matrix was normalized to the total thickness of the slice and the depth of cell penetration was plotted as the percent of the sample thickness.

### 2.10. Implantation of Decellularized Grafts for Immunological Evaluation

Decellularized facial grafts were implanted subcutaneously in immunocompetent C57BL/6 mice (female, mean weight 208 ± 15 g) to evaluate rejection and overall immune responses. Briefly, 5 mm biopsies from decellularized grafts were sterilized through incubation in 5% ethanol and 0.1% peracetic acid solution for 24 h under continuous shaking. The samples were extensively washed in sterile saline solution before implantation. For implantation, mice were anesthetized, laid in prone position, and a midline incision was made to create a subcutaneous pocket. One piece of sample was placed inside the pocket and the skin was closed using a resorbable suture. In addition to the decellularized sample, Permacol^TM^ (Medtronic, Minneapolis, MN, USA), a clinically used acellular porcine dermis, was implanted as a control group (n = 3). The implants were maintained in vivo for 21 days and were observed for clinical signs of rejection or inflammation. At the end of 3 weeks, animals were sacrificed, and spleens were collected for flow cytometry analysis of immune cells. Briefly, splenocytes were recovered and the cell concentration was determined using a cell counter. Moreover, 1 × 10^6^ cells per animal were incubated for 15 min at room temperature with fixable viability dye (eBioscience, ThermoFisher Scientific), washed twice in FACS buffer (PBS + 2% heat-inactivated fetal bovine serum), and labeled with fluoroconjugated antibodies. The samples were washed twice with FACS buffer and fixed with 2% paraformaldehyde for 20 min at room temperature and then washed and resuspended in FACS buffer. Cell populations were analyzed by flow cytometery using a BD FACSVerse (BD Biosciences, Woburn, MA, USA) and the data were analyzed using FlowJo software (version 10.6) (FlowJo LLC, Ashland, OR, USA). The following antibodies were used to identify T and B cell populations in splenocytes 21 days after sample implant:

Anti-mouse B220 (1:20, clone RA3-6B2 Biolegend, San Diego, CA, USA), anti-mouse CD3 (1:40, clone 17A2, Biolegend), anti-mouse CD4 (1:40, clone GK1.5, Biolegend), anti-mouse CD16 (1:10, clone S17014E, Biolegend), anti-mouse CD19 (1:80, clone 6D5, Biolegend), anti-mouse CD44 (1:80, clone IM7, Biolegend), anti-mouse CD62L (1:40, clone MEL-14, Biolegend), anti-mouse CD8a (1:80, clone 53-6.7, Biolegend), anti-mouse IgM (1:40, clone RMM-1, Biolegend), anti-mouse IgG (1:40, clone 115-605-164, Jackson ImmunoResearch, West Grove, PA, USA), and fixable viability dye (1:1000, eFluor 780, eBioscience, San Diego, CA, USA).

To determine changes in mouse T and B cell populations after 21-day implant of decellularized experimental and control tissues, a gating strategy was used. Briefly, live events negative for fixable viability dye in the lymphocyte singlet populations were analyzed. Total B cells were gated on CD3^−^/CD16^−^/CD19^+^/B220^+^ events. CD3^+^ T cells were gated on CD4^+^ and CD8^+^ events separately. T cell subsets were analyzed based on CD62L/CD44 expression where naive populations are CD62L^hi^CD44^lo^, effector memory CD62L^lo^CD44^hi^, and central memory are CD62L^hi^CD44^hi^.

### 2.11. Statistical Analysis

Graphical presentation and statistical analysis were performed on Prism 9 (GraphPad Software, La Jolla, CA, USA). For DNA, GAG, and collagen contents, unpaired Student’s *t*-tests were performed between native graft samples and decellularized matrix samples. For growth factors, two sample *t*-tests with Benjamini, Krieger, and Yekutieli correction were performed to quantitatively compare their abundance in native and decellularized tissues. For the immunological study, target cell populations were averaged across three mice per group and a one-way ANOVA test was performed to compare all experimental groups to the control Permacol™ implant group to see if there was a significant difference. Data are presented as mean ± SEM deviation for all analyses. A *p*-value less than 0.05 was considered statistically significant.

## 3. Results

### 3.1. Assessment of Decellularization Efficiency

**Figure 2:**
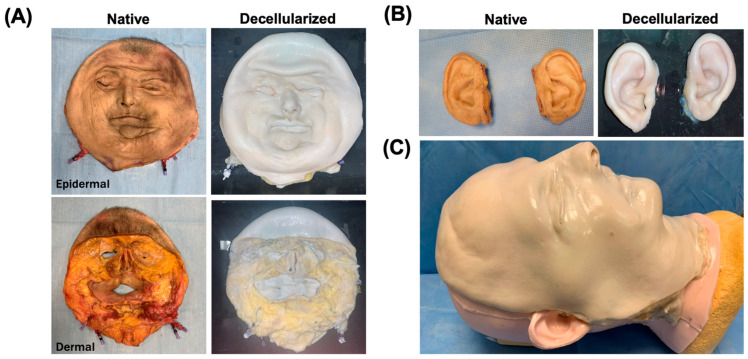
Macroscopic appearance of grafts before (native) and after (decellularized) decellularization from epidermal and dermal sides. (**A**) Facial graft. (**B**) Ear grafts. (**C**) Mock transplantation showing graft preserved morphology.

A total of three human facial grafts were decellularized through immersion in a series of decellularization solutions over a period of 15 days (Figure 1A). The grafts turned white at the end of the decellularization protocol, indicating successful removal of cells, while maintaining their physical shape. (Figure 2A). In addition to facial grafts, ears, composed of the skin and cartilage, from the same donor were also decellularized similarly. The ears also turned white, indicating successful decellularization, while maintaining their macroscopic appearance. (Figure 2B). A mock transplantation on a simulation model showed the preserved morphology of the grafts (Figure 2C).

**Figure 3:**
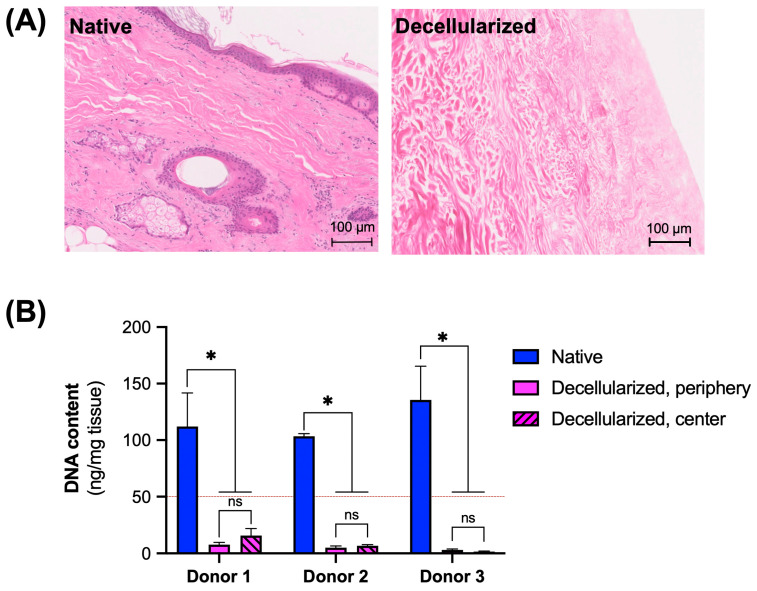
Removal of cells and DNA from grafts after decellularization. (**A**) Histological sections of native and decellularized flaps stained with H&E. (**B**) Quantification of DNA content in native and decellularized facial grafts. Biopsies were taken from two different locations, peripheral and center of skin, and analyzed separately. Dashed line shows 50 ng/mg threshold. * *p* < 0.05 by Dunnett’s multiple comparisons test of means. ns, not significant; n = 12 at least.

**Figure A2:**
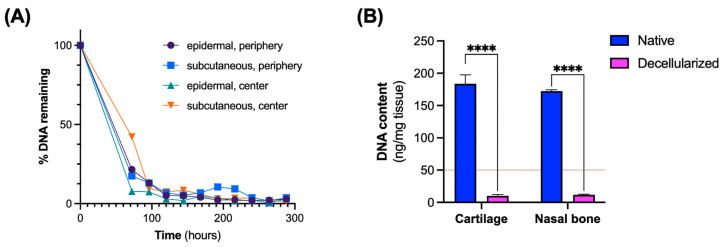
Decellularization efficiency of facial grafts. (**A**) Daily DNA quantification in facial graft during decellularization process. (**B**) Quantification of DNA content in native and decellularized ear cartilage and nasal bone tissue of grafts. Dashed line shows 50 ng/mg threshold, which is acceptable limit for complete decellularization. **** *p* < 0.0001 by Dunnett’s multiple comparisons test of means; n = 12 at least.

The successful removal of cells and cellular material from facial grafts was confirmed through H&E staining of histological sections, complemented by a quantitative analysis of DNA in the decellularized grafts. The absence of nuclei on H&E-stained sections indicated successful cell removal in the decellularized skin of the facial grafts when compared to native tissue (Figure 3A). The average DNA content was measured to be 117.0 ± 20.6 ng/mg and 6.7 ± 2.0 ng/mg tissue in native and decellularized facial grafts, respectively, indicating a 98.3% decrease in DNA content. The evaluation of the time course of DNA removal showed that decellularization was achieved (DNA content was less than 50 ng/mg of tissue) as early as 72 h of SDS exposure. Decellularization was consistent throughout the grafts, including both central and peripheral cutaneous and subcutaneous areas (Figure A2A). A statistically significant reduction in DNA content was observed between the decellularized and native samples across different donors. Furthermore, the DNA content of decellularized samples from the periphery and center of the grafts did not show statistically significant differences among donors (Figure 3B).

In addition to the skin, we also assessed nasal bone and ear cartilage samples for proper decellularization (Figure A2B). After decellularization, the DNA content of bone grafts from the helix, antitragus, concha, and bony regions was less than 50 ng/mg of tissue, representing a 93.2% reduction in DNA, a statistically significant difference. There was also a significant difference in DNA content between decellularized and native cartilage samples (*p* < 0.0001). The average DNA content of decellularized cartilage samples was 10.1 ± 10.1 ng/mg, corresponding to a 94.5% decrease compared to native samples.

### 3.2. Maintenance of Extracellular Matrix (ECM) Components

**Figure 4:**
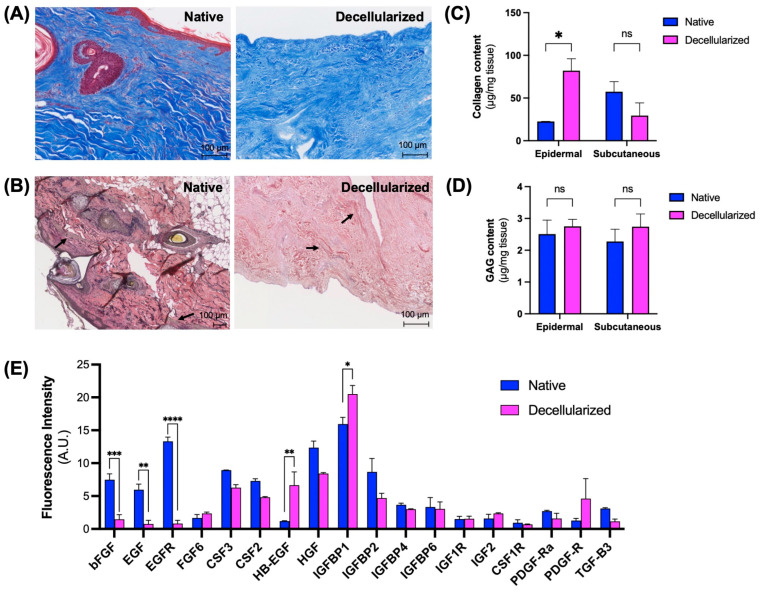
Extracellular matrix components in decellularized facial graft. (**A**) Masson’s Trichrome staining and (**B**) Miller’s (or Van Gieson) staining of native and decellularized grafts. Black arrows point to positive elastin staining. Biochemical analysis of (**C**) collagen and (**D**) GAG content in native and decellularized grafts. (**E**) Growth factor content in decellularized grafts compared to native tissue. ns: not significant; * *p* < 0.05, ** *p* < 0.01, *** *p* < 0.001, **** *p* < 0.0001 by Dunnett’s multiple comparisons test of means; n = 12 at least.

We analyzed the decellularized facial grafts for the remaining gross ECM components using histological staining and biochemical assays to confirm that the ECM composition is preserved after decellularization. In H&E-stained sections, the overall architecture of the ECM appeared well preserved after cell removal (Figure 3A). Major ECM components such as collagen and elastin were maintained as confirmed by Masson’s trichrome staining for collagen (Figure 4A) and Van Gieson’s staining for elastin (Figure 4B). Biochemically, collagen was found to be present in both the epidermal and the subcutaneous sides of the decellularized grafts; however, when compared to the native counterparts, the normalized collagen content was significantly higher on the epidermal side of the decellularized grafts (Figure 4C). There was no statistically significant difference in collagen content between the subcutaneous sections of the decellularized facial grafts and native tissue. Similarly, glycosaminoglycan content remained unchanged after decellularization, with no statistically significant differences observed across all analyzed sections (Figure 4D).

We compared the decellularized facial grafts to native tissues to assess the presence of bound growth factors and cytokines (Figure 4E). We found that following decellularization, the amount of basic fibroblast growth factor (bFGF), epidermal growth factor (EGF), and epidermal growth factor receptor (EGFR) was significantly decreased. On the other hand, heparin-binding EGF (HBEGF) and insulin like growth factor mRNA binding protein 1 (IGFBP1) were found to be significantly higher in decellularized grafts compared to native ones. All other tested factors were present in the decellularized grafts with no statistically significant differences.

### 3.3. Microarchitectural and Mechanical Characteristics of Decellularized Facial Grafts

**Figure 5:**
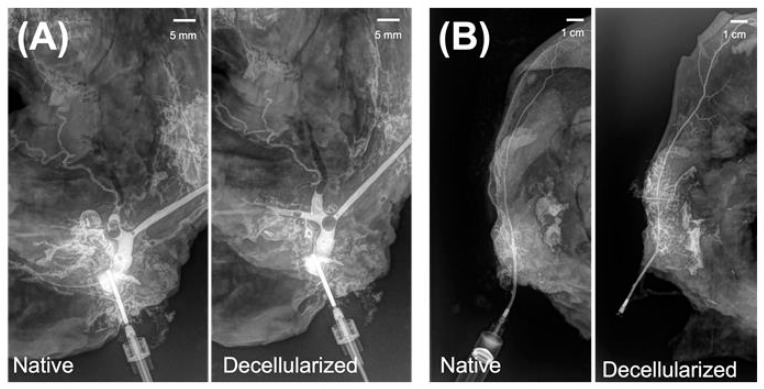
X-ray contrast angiogram of vascular network in native and decellularized face grafts. (**A**) Left facial artery. (**B**) Right temporal artery.

**Figure 6:**
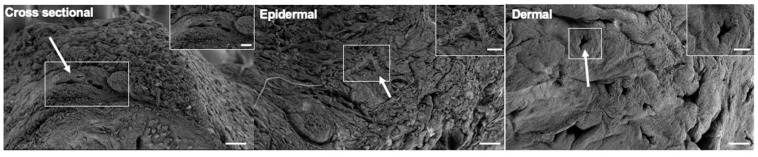
Scanning electron microscopy images of decellularized face samples; left to right: cross sectional, epidermal, and dermal views. White arrows indicate representative vascular structures in each image. Insets show magnified views of fields in white boxes. Scale bars 100 µm, insets 50 µm.

**Figure A3:**
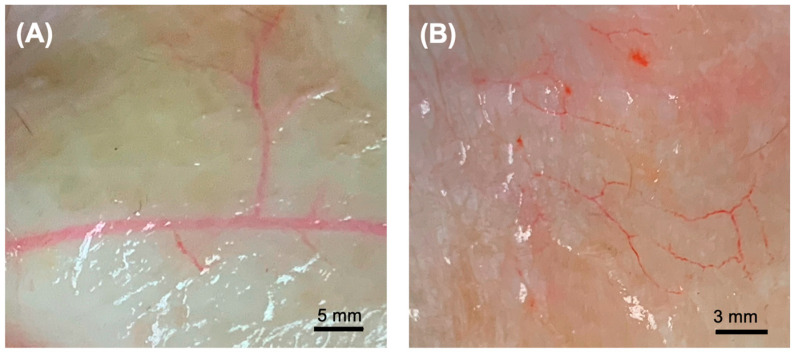
Vascular morphology after microfilm injection in decellularized facial grafts. (**A**) Frontal area. (**B**) Cheek area.

The vascular architecture of the grafts was imaged using X-ray angiography. Angiograms with the contrast agent injected into the left facial and the right temporal artery revealed no significant changes in the vascular architecture after decellularization when compared to the native grafts (Figure 5). Scanning electron microscopy of the decellularized facial grafts revealed well-preserved ultrastructural features of the ECM (Figure 6). We found the presence of vessels with sizes ranging between 30 and 100 µm (inset) in cross sectional images, and that the epidermal side of the grafts had rough but porous topology while the dermal side was smooth with larger openings. The corrosion cast of the decellularized grafts showed that the superficial capillaries were present (Figure A3).

**Figure 7:**
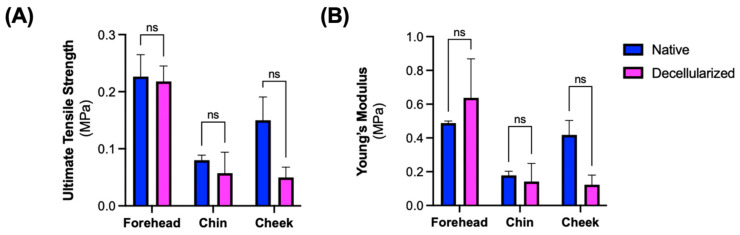
Tensile testing of decellularized facial grafts. (**A**) Ultimate tensile strength and (**B**) Young’s modulus of native and decellularized grafts sampled at three separate locations. ns: not significant; multiple paired *t*-tests with Holm–Šídák method.

Mechanical testing of the facial grafts revealed that the ultimate tensile strength and Young’s modulus of the decellularized samples were the same as native counterparts for the forehead and chin sections of the facial grafts. The strength and the modulus of elasticity were reduced in the cheek section of the facial grafts after decellularization although the differences were not found to be statistically significant (*p* = 0.0862 and 0.0561, respectively) (Figure 7A,B).

### 3.4. In Vitro and In Vivo Biocompatibility of Decellularized Facial Grafts

**Figure 8:**
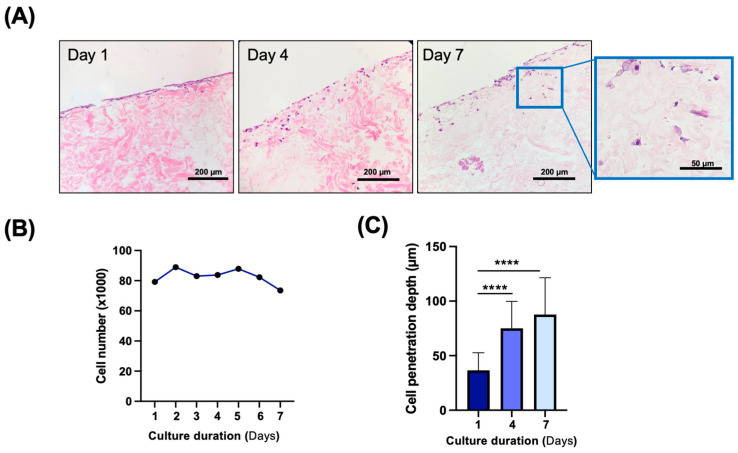
In vitro biocompatibility of decellularized facial grafts. (**A**) H&E staining showing cell attachment and penetration through dermal side of scaffold on days 1, 4, and 7 after cell seeding. Blue boxed section is shown to right at higher magnification to demonstrate cell morphology. (**B**) Cell proliferation as by Presto Blue assay. (**C**) Quantification of cell penetration depth as distance from the epidermal surface of scaffold over 7 days of culture. **** *p* < 0.0001 via Student’s test, n = 3. (Scale bars, main figure = 200 μm; scale bars, zoomed in image = 50 μm).

The decellularized facial grafts were tested for their biocompatibility through culturing human dermal fibroblasts on the dermal side of the full thickness sections over a period of seven days. The cells readily attached on the surface of the grafts on day one and progressively penetrated into the sections over the duration of the cultures (Figure 8A). The cell numbers were measured to be stable (Figure 8B) while the cells were found to engraft deeper into the scaffolds over the culture period (Figure 8C). Unfortunately, cells seeded on the epidermal side of the samples did not survive.

**Figure A4:**
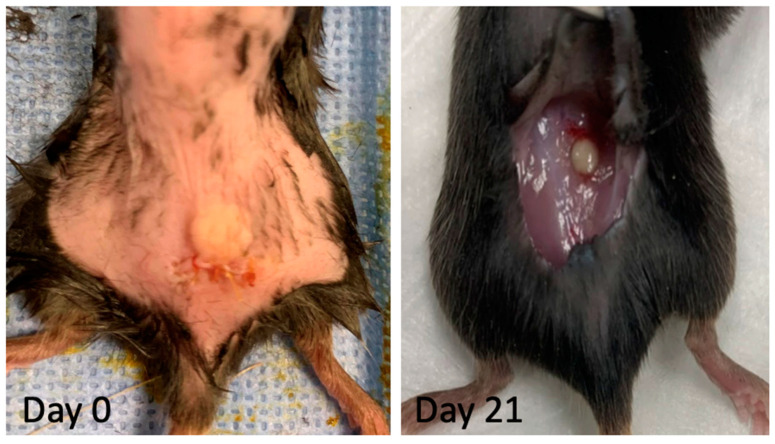
Implantation of decellularized facial grafts in mice. Macroscopic appearance of grafts in subcutaneous implantation site on day 0 and day 21 post implantation.

**Figure A5:**
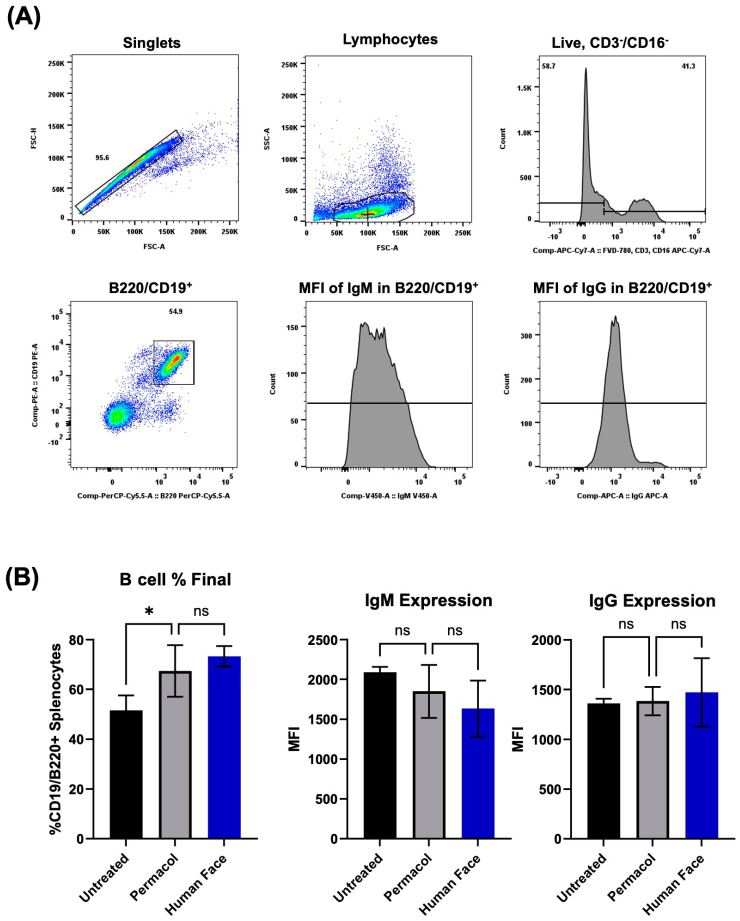
Flow cytometry analysis of B lymphocytes in spleen of mice implanted with decellularized facial grafts. (**A**) Gating of splenocytes moving from left to right down the figure. B cells were identified by gating on single viable cells that were CD3^−^/CD16^−^ and B220/CD19^+^. MFI of antibodies directed against IgM and IgG surface markers of B cells are shown. (**B**) Analysis of B cell % within viable CD3^−^/CD16^−^ events followed by IgM and IgG expression of B cells. ns = not significant, * *p* < 0.05, by one-way ANOVA tes.

**Figure A6:**
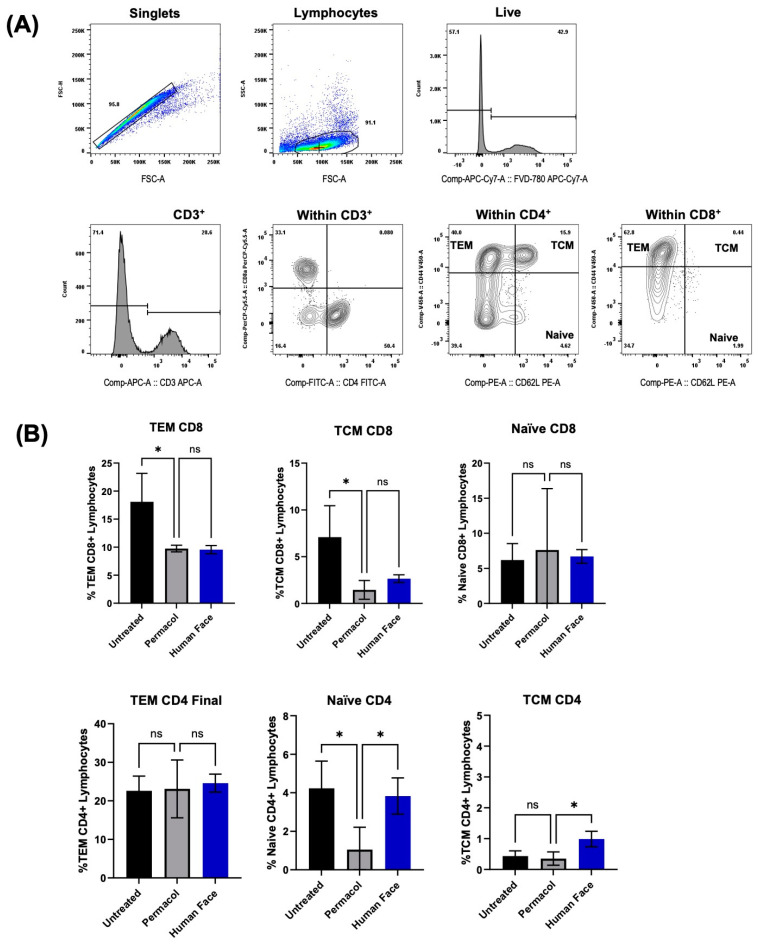
Flow cytometry analysis of T lymphocytes in spleen of mice implanted with decellularized facial grafts. (**A**) Gating of splenocytes moving from left to right down the figure. T cells were identified by gating on single viable cells that were CD3^+^. Within the CD3^+^ gate cells were separated into CD4^+^ or CD8^+^, followed by analysis of TEM, TCM, and Naïve lineages within each population based on expression of CD44 and CD62L surface markers. (**B**) Percentage of TEM, TCM, and Naïve cell lineages withing CD4^+^ or CD8^+^ splenocytes from each treatment group. ns = not significant, * *p* < 0.05, by one-way ANOVA test.

Upon implantation, the grafts remained intact with no clinical signs of inflammation at the end of 21 days (Figure A4). Spleens were harvested from implanted mice on day 21 to determine if T and B cell populations differed between decellularized facial tissue and Permacol controls (Figure A5 and Figure A6). Changes within these cell subsets could indicate in vivo immune sensitization to decellularized tissue throughout the protocol. Age matched untreated control mouse splenocytes were also analyzed for comparison. No significant differences were found in surface IgM or IgG expression of B cells between decellularized facial tissue and Permacol controls. T cell populations between these mice were also similar, with no significant changes in CD8^+^ effector memory, central memory, or naïve cells. The only differences noted were within CD4^+^ central memory and naïve populations, which both increased in experimental mice compared to the Permacol control group. However, these are minor immune cell subsets accounting for less than 5% of CD4^+^ lymphocytes isolated from the spleen.

## 4. Discussion

This report describes successful decellularization of human cadaveric face grafts by immersion only. Our novel protocol showed complete and homogeneous decellularization of whole face grafts and ears while maintaining their morphology, collagen, and GAG contents and allowing cell engraftment, creating scaffolds with preserved tensile strength that were non-immunogenic when transplanted to mice for 21 days.

Tissue or organ decellularization techniques involve immersing or perfusing with solutions containing chemical or biological agents, or applying physical stresses, to break down cell membranes and eliminate cells. The choice of perfusion versus immersion technique depends on the properties of the target tissue or organ including thickness, structure, and presence or level of vascularity [19]. Although perfusion decellularization of facial grafts have been shown before [15], immersion in detergents and other chemicals is a common technique of decellularizing dermal grafts [20,21,22]. In this study, we aimed to preserve the morphology and the microstructure of the face grafts better through immersing the grafts in detergent solution as opposed to using perfusion. We achieved the development of acellular matrices from human cadaver face grafts through immersion in a 1% SDS solution. In order to minimize the detrimental effects of high detergent concentration, we also tested immersion of a whole face graft in a 0.2% SDS solution following the same protocol; however, the resulting DNA content was found to be above the threshold of 50 ng/mg of tissue, thus, the protocol using 1% SDS was further analyzed. Immersion alone could be less aggressive on blood vessels, avoiding prolonged contact with detergents. This could eliminate the venous return problems where venous outflow is not observed potentially due to extensive damage to the vasculature of the decellularized graft [16]. Another important challenge when perfusion decellularizing tissues is to maintain stable, safe levels of pressure throughout the procedure [18]. Immersion of the graft prevents prolonged exposure of the pedicle to pressure, further minimizing the risk of vessel rupture. The X-ray angiography and corrosion cast of the decellularized grafts demonstrated that the vascular architecture remained intact with no significant damage to the arterial blood vessels.

Our protocol yielded whole face grafts with low DNA content and preserved collagen and GAG content consistently with all three donor tissues. In addition, the morphological characteristics of the epidermal and dermal ECM layers were preserved, i.e., the epidermal side was rough and the dermal side was smooth, suggesting that the high detergent concentration was not detrimental to the content or the organization of the scaffolds. Although we observed that bFGF, EGF, and EGFR were not preserved on the decellularized scaffolds, the majority of the growth factors were found at levels similar to those in native tissues. bFGF is an important factor in wound healing, angiogenesis, and skin regeneration, and is produced by skin dermal fibroblasts [23,24]. EGF and EFGR, produced by keratinocytes, work together where EGF binds to the EGFR, initiating a cascade of events essential for skin development, cell proliferation, wound healing, and the maintenance of skin homeostasis [25,26]. These crucial factors could potentially be externally supplemented prior to the cellularization of the graft, and recellularization with dermal fibroblasts and keratinocytes may alleviate any associated negative outcomes.

Decellularized grafts offer the advantage of providing cells with access to native ECM structures, which leads to improved tissue integration and function. In order to render these scaffolds transplantable, recellularization is crucial. Our results showed that human dermal fibroblasts, when seeded on the dermal side of the grafts, can attach, maintain viability, and penetrate to the deeper sections of the tissue. This preliminary recellularization experiment showed that the scaffolds are not cytotoxic. Recellularization of the whole graft, through injection or perfusion using a bioreactor, as well as endothelization of the microvessels prior to transplantation, will be important future work. It is important to note that only seeding from the dermal side of the sample enabled cell attachment. This could be solely related to the fact that we used a small skin sample for this preliminary recellularization experiment, and the samples were afloat when cell seeding was conducted. The more closed surface structure of the epidermis and the small size of the tissue used for this seeding could explain the lack of cell attachment on the epidermal side. The use of larger scaffold sections for epidermal seeding should be tested for further conclusions.

In addition to the in vitro biocompatibility, we tested the immunogenicity of decellularized face grafts through implanting a small section of the resulting scaffold into mice. Compared to a commercially available soft tissue surgical implant Permacol, the decellularized face grafts did not cause a higher percentage of B cells that express CD19 and B220 surface antigens, suggesting no immune response in mice. This was supported by the visual observations of the mice and the scaffolds after 21 days of implantation. In addition, IgM and IgG expressions were not found to be increased in mice implanted with face grafts, similar to the Permacol controls and untreated mice. This suggests that neither early nor late stage immune response was present in mice after 21 days [27,28]. In line with these results, we did not observe any difference in percent CD8^+^ and CD4^+^ naïve or effector memory T cells between the untreated, Permacol-treated, and decellularized graft implanted mice. Although the graft did not cause any change in percent CD8^+^ central memory T cells, it led to a significant increase in CD4^+^ central memory T cells. During decellularization, some antigens, such as major histocompatibility complex (MHC) molecules and minor histocompatibility antigens, can remain within the decellularized extracellular matrix [29]. The release of damage-associated molecular patterns (DAMPs) during this process triggers an increased expression of MHC II and co-stimulatory molecules on the recipient’s antigen-presenting cells (APCs), thereby enhancing T cell recognition of these remaining antigens. MHC II was shown to specifically stimulate CD4^+^ T cells [30]; thus, this may suggest a mild immune reaction in mice. The lack of increase in other T cell or B cell populations, however, indicates in vivo biocompatibility. In order to strengthen our conclusion on the immunogenicity of decellularized face grafts, two additional control groups can be tested; a “mock” group, in which we would simply make skin incisions, followed by blunt dissection of the subcutaneous tissue, without implanting any material, and a group with implantation of native human graft tissue which would serve as a positive control for observing immune reaction. In addition, future studies will use an increased number of mice per group and will perform phenotyping of the circulating blood cells (by including myeloid lineage markers and intracellular cytokine staining). Overall, these improvements will enable validation of the results found on T lymphocyte response with Permacol™ (CD4) and face samples (CD8).

While no other reports of immersion-decellularized full face grafts were found, many other small specimens suitable for reconstruction and transplantation have been decellularized by immersion. For example, Sano et al. proposed the use of decellularized human adipose tissue as an injectable tissue filler for reconstruction, suggesting that it may be useful for enhancing soft tissue volume and shape in reconstructive surgeries without presenting a risk of immune rejection [31]. In addition, to address the current challenge of areolar and nipple complex reconstruction in breast cancer patients, for which options are limited and results variable, we have developed a protocol for the decellularization of large animal nipples through immersion [32]. Decellularization of more complex tissues has been shown using a combination of perfusion and immersion techniques. In 2019, decellularization of a human penis specimen produced a complete acellular penis matrix in 14 days, using a combination of micro-arterial perfusion, urethral catheter perfusion, and external diffusion of 1% SDS. The procedure preserved the vascular networks and morphology of the native tissue [33]. Another study showed the use of a combination of perfusion and immersion for decellularization of a complete cadaveric human upper limb [34]. The limb was perfused through the brachial artery while being immersed in detergent solution in a bioreactor. Decellularization was achieved using 1% SDS solution for 30 days followed by 1% Triton-X solution for 15 days. This long exposure to SDS, however, rekindles the problem of graft alteration due to prolonged exposure to detergents.

## 5. Conclusions

In summary, we demonstrated that decellularization is a highly advantageous method for developing complex tissue/organ grafts with natural 3D ECM. However, due to the inherent heterogeneity of different tissues, there is no standardized method for decellularization and each tissue type requires individual optimization. Due to the limited inclusion criteria for FVCA, decellularized face grafts provide a promising alternative as they can be recellularized with patient-specific cells, eliminating the risks associated with lifelong immunosuppressant therapy. Through this study, we present a novel and effective approach for decellularizing human cadaveric face grafts through immersion in detergent solution and demonstrate successful preservation of tissue morphology, ECM components, and tensile strength. The grafts allowed for cell attachment and penetration through the dermal side, showing in vitro biocompatibility. The immunogenicity of the grafts was also shown to be promising, although further analysis would be needed to confirm their suitability for transplantation. Overall, the protocol described here offers a robust, reproducible approach to decellularizing facial tissues, with potential applications in facial reconstruction. Further investigations into the patient-specific recellularization process are critical as the graft would not function without the cells. Additional work will also include the optimization of growth factor supplementation and cryopreservation strategies, which will give access to grafting materials readily available to rapidly repair complex facial skin defects.

## Data Availability

Data can be provided by the corresponding authors on demand.

## Abbreviations

The following abbreviations are used in this manuscript:

APCs	Antigen-presenting cells
bFGF	Basic fibroblast growth gactor
DAMPs	Damage-associated molecular patterns
dH_2_O	Deionized water
ECM	Extracellular matrix
EGF	Epidermal growth factor
EGFR	Epidermal growth factor receptor
FVCA	Facial vascularized composite allotransplantation
GAG	Glycosaminoglycan
HBEGF	Heparin-binding EGF
H&E	Hematoxylin and Eosin
IGFBP1	Insulin like growth factor mRNA binding protein 1
IIAM	International Institute for the Advancement of Medicine
IRB	Institutional Review Board
MGH	Massachusetts General Hospital
MHC	Major histocompatibility complex
PBS	Phosphate-buffered saline
SDS	Sodium dodecyl sulfate
VCA	Vascularized composite allotransplantation

## Appendix A

**Table A1:** Checklist for face graft recovery surgery.

Donor Face Preparation	
1	Mark the donor face (coronal/pretragus/taking the cervical skin laterally)
2	Make incision starting from the neck to harvest the vessels at their origin
3	Localize the sternocleidomastoid muscle (SCM)
4	Expose the external jugular vein (EJV)
5	Free up the SCM laterally to expose the vessels
6	Dissect the external carotid and the internal jugular vein (TLF trunk)
7	Locate the division of the digastric muscle and hypoglossal
8	Section the posterior belly of the digastric muscle
9	Localize the hypoglossal nerve (source of nerve graft if needed)
10	Expose the facial nerve at its origin (retro auricular) to obtain the maximum length
11	+/− Transect the external auditory canal (more length for the facial nerve) and dissect the maxillary artery
12	Ligate the posterior maxillary artery
13	Make incision of the scalp (coronal incision)
14	Raise the scalp in a sus periosteal plane up to 1 cm before the appearance of the supra orbital nerve and then in a subperiosteal plane
15	Cut the supraorbital nerveas close to the bony foramen
16	Localize the Levator palpabrae superioris (LPS) muscle (with the eyeball in place++ to facilitate the location of the LPS), test it, and tag it using a stitch
17	Perform an enucleation and keep the maximum of conjunctiva (circular incision)
18	Cut off the superior eyelid horizontally
19	Cut off the inferior eyelid (with the maximum amount of conjunctiva): dissect through the inferior fornix to just inside the inferior orbital rim, which will allow dissection through the inferior orbital fat and lower lid retractors, preserving the palpebral conjunctiva
20	Localize and cut the inferior orbital nerve
21	Raise the parotid and expose the Mandible
22	Stay on the top of the masseter muscle (safe plane)
23	Identify the buccal fat pad at the anterior border of the masseter. The buccal fat pad is left in place
24	Finish up the dissection to the orbit and the zygoma
25	Perform osteotomy of the nasal bone and continue laterally to include part of the maxillary bone
26	Cut off the nasal septum longitudinally
27	Cut off the superior mucosa gingiva
28	Cut off the inferior mucosa gingiva
29	Cut off the mental nerve as close to the bony foramen
30	Connect the planes in the middle to connect to the oral cavity (on top of the sternohyoid and omohyoid superior muscle +/− anterior jugular vein)
31	Ligate the internal/external jugular vein/external carotid as far as possible
32	Detach entirely the face
33	Wash the free flap with heparin saline and check the integrity of the facial artery
34	Place the face in an organ preservation solution (static cold storage)
